# Grain Refinement of a Powder Nickel-Base Superalloy Using Hot Deformation and Slow-Cooling

**DOI:** 10.3390/ma11101978

**Published:** 2018-10-14

**Authors:** Xianqiang Fan, Zhipeng Guo, Xiaofeng Wang, Jie Yang, Jinwen Zou

**Affiliations:** 1 School of Materials Science and Engineering, Tsinghua University, Beijing 100084, China; fxq16@mails.tsinghua.edu.cn; 2 Laboratory for Advanced Materials Processing Technology, Ministry of Education, Tsinghua University, Beijing 100084, China; 3 AECC Beijing Institute of Aeronautical Materials, Beijing 100084, China; wangxiaofeng_0404@163.com (X.W.); yangjient@163.com (J.Y.); jinwen_zou@126.com (J.Z.); 4 Science and Technology on Advanced High Temperature Structural Materials Laboratory, Beijing Institute of Aeronautical Materials, Beijing 100084, China

**Keywords:** nickel-base superalloy, hot deformation, cooling, twining, γ’ precipitates

## Abstract

A pre-hot-deformation process was applied for a polycrystalline nickel-base superalloy to active deformation twins and dislocations, and subsequent slow cooling treatment was used to achieve grain refinement and microstructure homogenization. The microstructural evolution of the alloy was investigated, and the corresponding underlying mechanism was discussed. It was found that twinning mainly occurred in large grains during pre-hot-deformation owing to the stress concentration surrounding the large grains. High density dislocations were found in large grains, and the dislocation density increased approaching the grain boundary. The average grain size was refined from 30 μm to 13 μm after slow cooling with a standard deviation of grain size decreasing from 10.8 to 2.8, indicating a homogeneous microstructure. The grain refinement and microstructure homogenization during cooling process could be achieved via (i) static recrystallization (SRX), (ii) interaction of twin tips and γ’ precipitates, and (iii) grain coarsening hindered by γ’ precipitates in grain boundaries.

## 1. Introduction

Polycrystalline nickel-based superalloys are widely used in the hot sections of modern aero-engines and gas turbines due to excellent properties including high strength, corrosion resistance, and fatigue resistance at elevated temperature [1,2,3,4,5]. The mechanical property is controlled by grain structure formed during hot deformation [6,7,8] and the distribution of γ’ precipitate achieved during the cooling process [9,10,11]. As high temperature industries are continuously requiring superalloy with even higher strength, superalloys with complex chemical composition containing heavy elements have been developed, which in turn has led to the difficulties of achieving segregation-free, homogenous superalloys via traditional ingot metallurgy [12]. The difficulties have been settled by employing hot isostatic pressing (HIP) followed by isothermal forging [12,13].

It is well known that the microstructure evolution during hot deformation is critical in determining the final microstructure. In particular, both dynamic recrystallization and twining play an important role for grain refinement and microstructure homogeneity [14,15], which in turn could improve the workability and mechanical properties for the alloy [16,17,18]. During hot deformation before cooling, both the microstructure and the stress-strain evolution are highly dependent on the employed hot deformation parameters including temperature [19,20] and strain [19,21,22].

Ning et al. [15] found that the twin boundaries became the DRX preferred nucleation sites for a HIP superalloy FGH4096. Chen et al. [23] studied the microstructural evolution and deformation features of a nickel-based superalloy and found that the evolution pattern of dislocation substructure was from high dislocation density to dislocation network and finally to subgrain to DRX grain. Kumar et al. [24] studied the effect of strain rate on the microstructural evolution during hot deformation of a HIP nickel-base superalloy and found that high strain rate could promote the evolution of twin boundaries during hot deformation. Zhang et al. [25] investigated the dynamic recrystallization behavior of a nickel–base alloy and found that the nucleation of DRX could also be activated by the twinning formation. Moreover, the strengthening effects associated with a high number of twin boundaries for alloys like steel, nickel, and copper have been reported in literatures [18,26]. It is essential for nickel-based superalloys used in aero-engines, because the improvement of corrosion and fatigue resistance can be achieved via increasing twin boundary fraction without alteration of the chemical composition of the alloy. Previous works showed that compared with other high-angle grain boundaries, twin boundaries had low interfacial energy and acted as a barrier for dislocation slip during plastic deformation [27,28]. Accordingly, the presence of the twin boundaries improved mechanical properties of the materials [29].

Beside hot deformation, the microstructural evolution during cooling after pre-hot-deformation is also essential for determining the final microstructure. Both SRX and γ’ precipitation occurred during cooling after hot deformation. Dislocations formed via pre-hot-deformation promoted the precipitation of γ’ during subsequent cooling [30,31]. On the other hand, coherent γ’ precipitates could hinder the motion of dislocations [32] and slows the nucleation of the SRXed grains during cooling. However, the exact evolution pattern of microstructure during cooling process is far from clear, and very limited studies have been performed to study it.

The objective of the present work is to understand the mechanism of the microstructural evolution of a powder metallurgy superalloy during cooling process. The evolution of grain size and twin boundary was determined and correlated to the microstructure patterns. In present work, heavy deformation was applied at elevated temperatures prior to the slow cooling process to achieve fine and homogeneous grains, with particular attention paid to twining during pre-hot-deformation.

## 2. Materials and Methods

### 2.1. Material

The material used in this study is a polycrystalline nickel-based superalloy, namely, FGH96. The alloy was prepared using hot isostatic pressure (HIP) and then processed using a two-step method. The two-step method comprised hot deformation after HIP and a subsequent slow-cooling. The FGH96 powder was produced via argon atomization after melting the ingot using vacuum induction heating. The powder was then sealed into a stainless steel container and HIP consolidated at a temperature lower than the γ’ solvus temperature. Table 1 shows the nominal composition of the material.

### 2.2. Pre-Hot-Deformation and Subsequent Cooling

Four cylindrical rods of 9 mm in diameter and 14 mm in length were designated as specimen A, B, C, and D, respectively. These specimens were extracted from the billet after HIP for hot deformation. To eliminate the effect of γ’ precipitate on hot deformation, specimen A, B, C, and D were rapidly heated to 1150 °C and holding for 30 min to dissolve all γ’ precipitates [33] prior to hot deformation using a Gleeble 1500 thermal simulator system. Without any deformation, specimen A was used as a blank control contrast group.

Specimens B and C were then compressed to a strain of 0.2 and 0.5, respectively, at 1150 °C with a strain rate of 0.01 s^−1^. After dissolving γ’ precipitates, specimen D was first compressed to a strain of 0.5 at 1150 °C with a strain rate of 0.01 s^−1^ and slowly cooled to 1000 °C at a cooling rate of 6 °C/min. To retain corresponding elevated temperature microstructure, all the samples were water quenched after hot deformation or cooling.

### 2.3. Microstructural Characterization

The microstructure on the sectioning planes perpendicular to the loading axis was characterized using electron backscatter diffraction (EBSD, ZEISS Merlin, Heidenheim, Germany) and transmission electron microscopy (TEM, JEOL 2100 F, Tokyo, Japan). The samples for EBSD analysis were prepared by grinding with a 4000 SiC paper and electrochemical polishing at 30 V for 20 s in the electrolyte of 20% hydrochloric and 80% methyl alcohol at room temperature. The EBSD scanning parameters were set as 2 μm step length for 400 μm × 400 μm scanning area and 0.5 μm step length for 80 μm × 80 μm scanning area. The specimens for TEM were mechanically polished to ~80 μm and further thinned by ion milling with a voltage of 6 kV. The thickness of the final thin TEM sample was about 100 nm–200 nm. The EBSD data was analyzed using Oxford HKL Channel 5 software (Oxford, UK).

## 3. Results

### 3.1. Mechanical Performance

**Figure 1:**
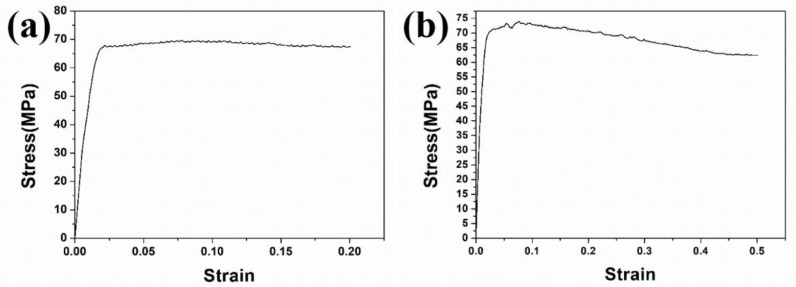
Typical stress-strain curve obtained at a strain rate of 0.01 s^−1^ at 1150 °C to a strain of (**a**) 0.2 and (**b**) 0.5, respectively.

Figure 1a shows a typical stress-strain curve of the hot deformation of specimen B to a strain of 0.2 at a strain rate of 0.01 s^−1^ at 1150 °C. It is clear to observe a steady stress after yielding at 68 MPa at ε = 0.02, which maintained even to a strain of 0.2, indicating the retention of dynamic equilibrium between working hardening and high-temperature softening. Figure 1b shows the stress-strain hot deformation curve of specimen C at 1150 °C to a strain of 0.5 at a strain rate of 0.01 s^−1^. After peaking at ~72 MPa, the curve of specimen C shows a narrow plateau followed by 14% drop at ε = 0.5, suggesting the occurrence of the overwhelming softening after strain of 0.2.

### 3.2. Microstructural Evolution during Hot Deformation

**Figure 2:**
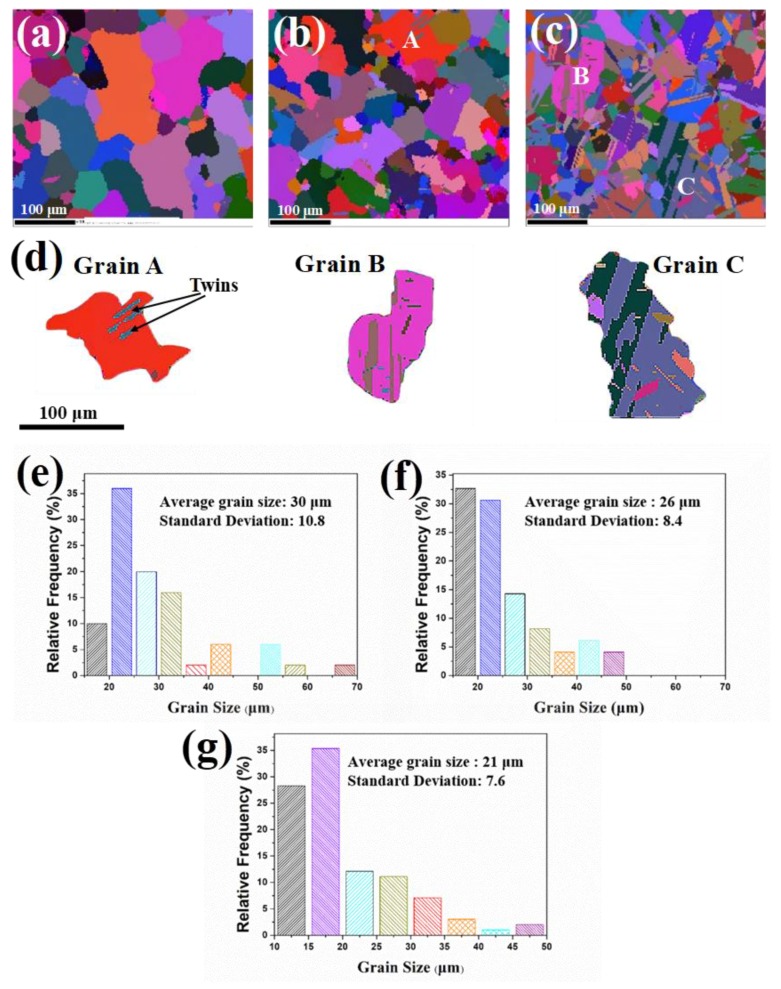
Euler angle map after the pre-hot-deformation with a strain of (**a**) 0, as blank control group, (**b**) 0.2, and (**c**) 0.5; (**d**) shows the large grains of A, B, and C extracted from (**b**,**c**); (**e**–**g**) are the corresponding grain size distributions of (**a**–**c**).

Figure 2 shows the Euler maps and the corresponding grain size distribution of the samples after hot deformation to a strain of 0, 0.2, and 0.5 at 1150 °C (with a strain rate of 0.01 s^−1^). After hot deformation to a strain of 0.5, the microstructure evolved from coarse grains to finer and more homogeneous twinning grains. 

Figure 2b,f shows that after hot deformation to a strain of 0.2, the average grain size was 25 μm, i.e., a decrease by 13.8% from that of the as-HIP sample (see Figure 2a,e). However, DRX grains is not exhibited in Figure 2b, indicating the softening effect could be mainly attributed to dynamic recovery (DRV, i.e., the initial stage of DRX) before the strain approached 0.2. Except for grain A, which was one of the largest grains in Figure 2b, almost no twins exhibited in Figure 2b, indicating slipping was the deformation pattern in most grains before the strain of 0.2. Accordingly, the transition of deformation pattern from slipping to twinning occurred in large grains after hot deformation to a strain of 0.2.

The grains were further refined by applying a strain of 0.5, and the average grain size became 20 μm, i.e., a 30.4% decrease compared with that of the as-HIP sample (see Figure 2c,g). Figure 2c shows that twins propagated significantly as the strain reached 0.5, and the fraction of twin boundary was determined to be 58% at a strain of 0.5. However, similar to the microstructure subjected to smaller strain, twins again mainly existed in large grains with increasing quantity (see Figure 2c). Typical large grains labelled as A, B, and C in Figure 2b,c were depicted in Figure 2d for detailed observations. Furthermore, standard deviation of grain size was utilized to describe the degree of homogeneous of grain size. The standard deviation decreased from 10 in as-HIP sample to 7 for hot deformation to a strain of 0.5, indicating the grain size tended to become homogeneous during hot deformation.

### 3.3. Microstructural Features after Slow Cooling

**Figure 3:**
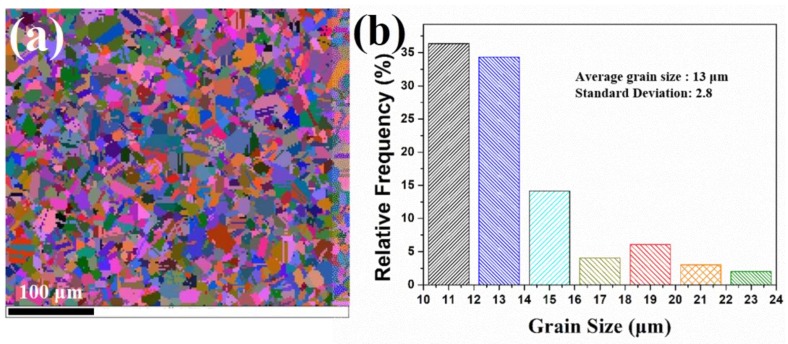
(**a**) Euler angle map after cooling to 1000 °C with a cooling rate of 6 °C /min. (**b**) The grain size distribution of (**a**).

**Figure 4:**
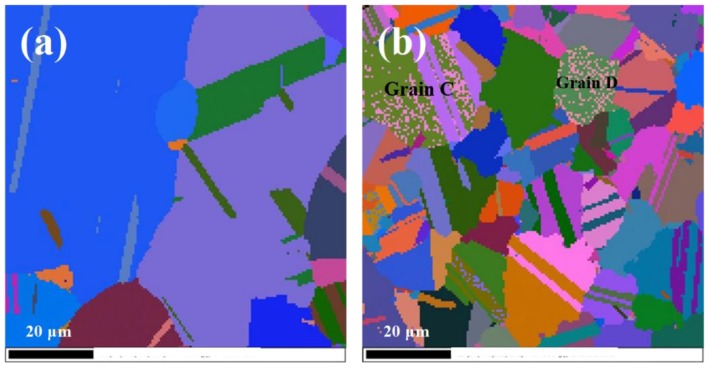
Euler map after hot deformation at 1150 °C to a strain of (**a**) 0.5 and (**b**) cooling to 1000 °C with cooling rate of 6 °C /min.

Figure 3a shows the significant change of microstructure after slow cooling (cooling rate was 6 °C/min). Figure 3b shows that as cooling to 1000 °C the average grain size was further refined to 13 μm with a standard deviation of 2.8. Figure 3a was enlarged and shown in Figure 4b to achieve a better observation of the microstructural details of the slow-cooled sample. More twins were activated during slow cooling, because almost all refined grains had twins. Figure 4b shows a large number of ultra-fine grains (colored by pink) exhibited in grains C and D (in the Euler map), indicating the occurrence of continuous dynamic recrystallization (CDRX). For CDRX, new grains formed by the progressive rotation of subgrains, resulting in an increase of their misorientation angles, which usually occurred in the interior of grains [34]. The main feature of twin lamellae was that the twin boundaries passed through the entire grain during the cooling process. In other words, after slowly cooling to 1000 °C, there exhibited almost no twin tips inside the grains.

## 4. Discussion

### 4.1. Twined Grains and Twining Mechanism during Hot Deformation

Slipping and twining are the main deformation mechanisms in alloys. Selection of deformation pattern is mainly determined by two factors, i.e., Schmid factor (*SF*) and critical resolved shear stress (*CRSS*), whose relation is

(1) SF=cosλcosφ

(2) CRSS=SFσ

in which σ is the loading stress, λ is the angle between the load axis and slipping direction, and φ is the angle between the load axis and the normal of the slip plane. The compression direction was perpendicular to the observation plane; accordingly, the Schmid factor for the twelve fcc slip systems in grain A was calculated and listed in Table 2.

**Figure 5:**
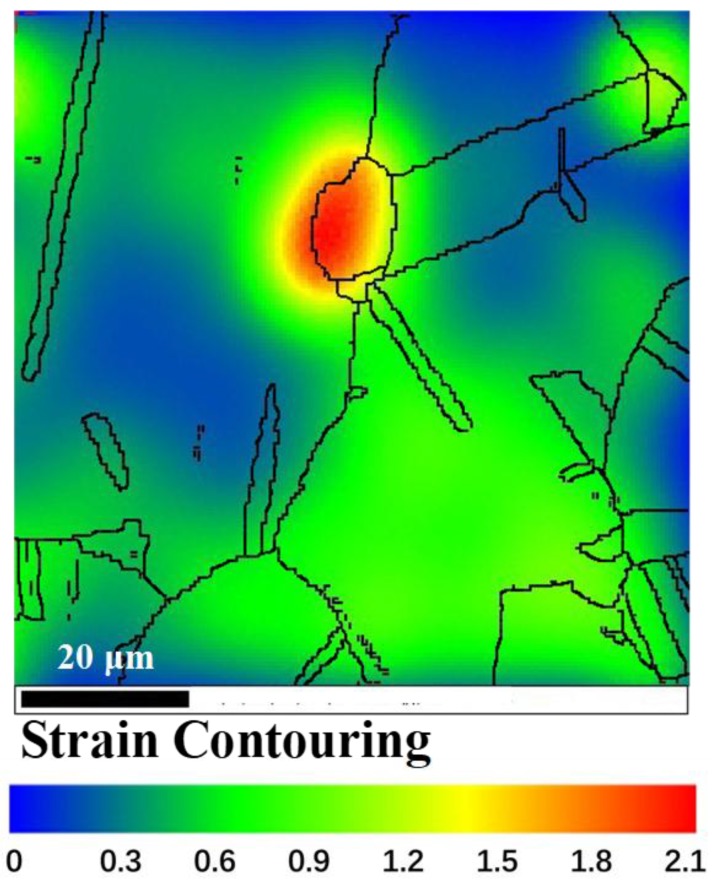
Strain distribution after hot deformation to a strain of 0.5 at a strain rate of 0.01 s^−1^ at 1150 °C.

It is shown that [$10\bar{1}$](111) and [101]($1\bar{1}\bar{1}$) were the easiest starting slip systems with a Schmid factor of 0.49, very close to the upper limit of 0.5. Accordingly, if loading stress was the only applied stress on grain A, the slip deformation should be the only deformation mode, and no twining occurred. However, several parallel twins were exhibited in grain A (see Figure 2d). The twinning deformation must be attributed to microstructure heterogeneity, which could lead to high stress concentrations. The magnitude of stress concentration and the twining deformation varied with the microstructure heterogeneity, including grain size distribution and misorientation of grain boundaries between adjacent grains. Figure 5 shows plastic strain distribution after hot deformation to a strain of 0.5. The strain in the specific grain boundaries was much larger than that of the intragranular region. The larger stress concentration promoted twining in these regions. It can be seen from Figure 2b,c that twins mainly existed in large grains. This is because that large grains were more easily subjected to stress concentration due to longer grain boundaries. Once the local shear stress exceeded certain critical value, the twinning formation occurred.

### 4.2. The Mechanism of the Microstructural Evolution during Cooling Process

Grain refinement and homogenization, as well as twinning propagation, were achieved during cooling. To understand the underlying mechanism driving this microstructural evolution, TEM images were obtained to exhibit the microstructure of specimen C and D.

#### 4.2.1. The Role of Static Recrystallization (SRX) in Grain Refinement

**Figure 6:**
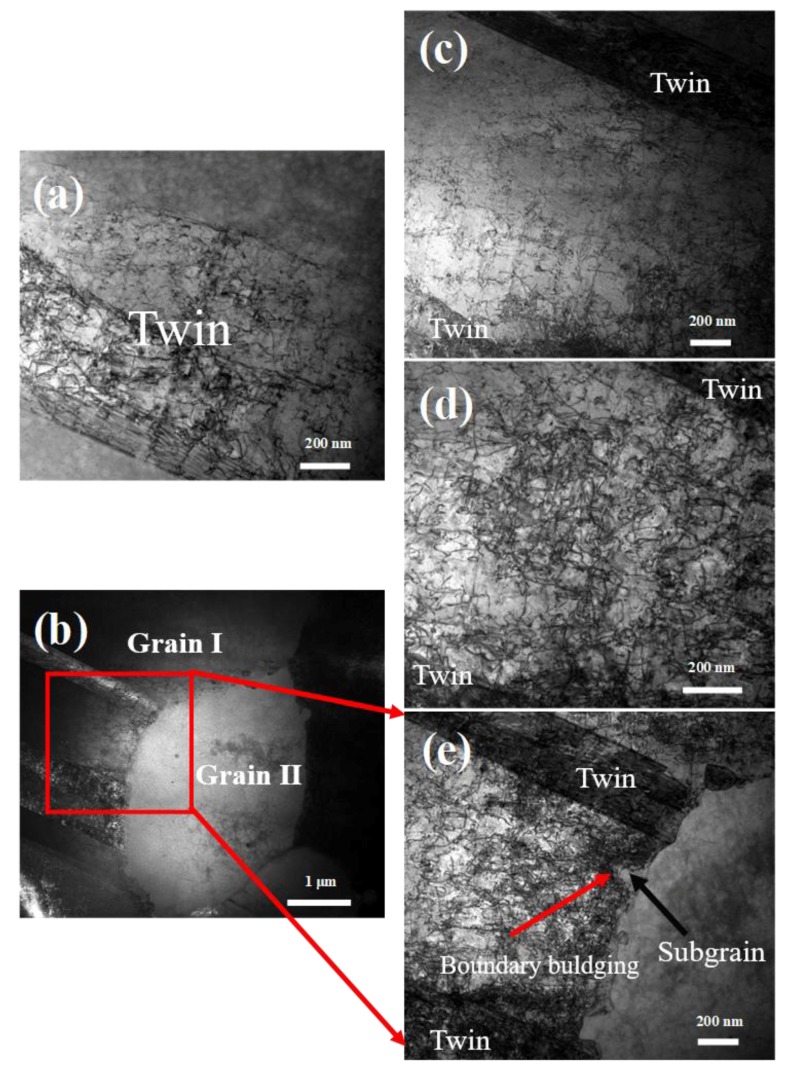
TEM images of FGH96 alloy after hot deformation at 1150 °C to a strain of 0.5; (**a**) dislocation distribution inside the twins; (**b**) two different size grains (grain I and grain II); (**c**) dislocation distribution inside the grain Ι; (**d**) dislocation distribution close to grain Ι boundary; (**e**) is a magnification of the region in (**b**) marked by red rectangular.

Figure 6 shows the initial stage of microstructural evolution during cooling process after pre-hot-deformation to a strain of 0.5. Figure 6c–e shows that dislocation density experienced a gradual increase approaching the grain boundary. This can be understood by investigating the facts that deformation occurred more significantly near grain boundaries and dislocation migrated toward grain boundaries during hot deformation. However, Figure 6b and Figure 6e shows that grain II, with a diameter of 2.5 μm that was much smaller than its average grain size as well as that of grain I, barely exhibited any dislocation. The significant difference of dislocation density led to a significant energy difference between the two adjacent grains. Generally, grain boundary bulging is associated with strain-induced grain boundary migration (SIGBM) [35], which is driven by the energy difference between the adjacent grains. In this respect, the boundary of those two grains was the preferred nucleation site for static recrystallization during subsequent cooling. From Figure 6e, it can be seen that there was a dislocation-free region, i.e., subgrain, behind the grain boundary bulging, which was the SRX nuclei with a radius [36]:

(3) $$R=-\frac{2\gamma }{\Delta E_{V}}$$

in which γ is grain boundary energy inhibiting the bulging of the grain boundary and $\Delta E_{V}$ is the strain energy of the deformed region acting as a driving force for SRX process. The subgrain could bulge into the matrix as a new grain when the strain energy of the deformed matrix was large enough to overcome the boundary energy of the SRX grain. It is well known that nucleation of SRX often initiated from the pre-existing boundaries and gradually expanded high-strain energy grain by absorbing dislocations. The subgrain depicted in Figure 6e bulged into a region with the highest dislocation density, implying the transformation of the large grain into plenty of SRX grains during the subsequent cooling process.

According to Figure 6a, plenty of dislocations accumulated in the twins after hot deformation to a strain of 0.5. Since the slip transfer across the twin boundaries was extremely difficult due to low interface energy [37,38,39], the dislocation accumulation at twin boundaries eventually transferred the twin boundaries into high angle grain boundaries (HAGBs) [37], which improved grain refinement. Besides, twins could further induce high dislocation density in large grains and make large grains become the preferred sites for SRX grain bulging. Accordingly, grain refinement and homogenization were achieved simultaneously after cooling.

#### 4.2.2. Contributions from Interaction of Twin Tips and γ’ Precipitate

The growth of γ’ precipitates, together with the propagation of twin boundaries during slow cooling, led to the interaction of γ’ precipitates and twin tips. The amount of the twin boundaries increased as cooling to 1000 °C (see Figure 4), indicating that there was a driving force for the propagation and extending of twin boundaries during the slowly cooling (at a cooling rate of 6 °C/s). It is well known that a high number of twin boundaries can significantly improve the mechanical properties of nickel-base alloy [40,41]. This is of importance for nickel-base superalloy, since it can improve the mechanical properties without altering the chemical composition of the alloy. 

**Figure 7:**
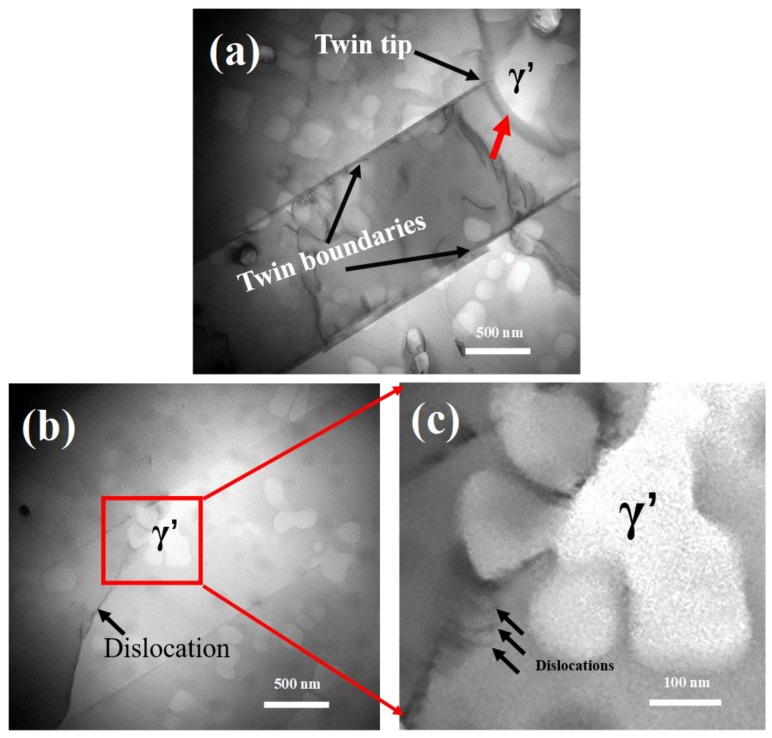
TEM images of FGH96 alloy after cooling to 1000 °C with cooling rate of 6 °C/min (**a**) the interaction of the twin tip and the γ’ precipitate; (**b**) the interaction of the dislocation and γ’ precipitate; (**c**) a magnification of the region in (**b**) marked by red rectangular.

The interaction of twin boundaries and γ’ precipitates played an important role in the grain refinement during slowly cooling. As shown in Figure 7a, the twin tip and the γ’ precipitate met in the interior of a grain, and the twin tip was unable to overpass the γ’ precipitate, indicating that γ’ precipitate could act as barrier for twin tip extending. Besides, it can be seen from Figure 7a that a dark contrast strip marked by red arrow existed in the vicinity of the γ/γ’ interface, indicating the rotation of the γ’ precipitate due to intensive interaction between the twin tip and the γ’ precipitate. The concomitant rotation of precipitate during cooling continuously changed the orientation of existing γ/γ’ interface, which eventually transformed into mobile HAGBs and further improved the grain refinement.

Twin tips ended up at those newly formed HAGBs, leading to the absence of twin tips inside the grains (see Figure 4b). Accordingly, except for twin’s direct extending to the pre-existing grain boundaries, the rotation of the γ’ precipitate induced by the interaction of twin tip and γ’ precipitate also contributed to the absence of twin tips inside the grains. Because the stress concentration was generally observed at twin tips [42], which was harmful for the stable mechanical properties of the alloy, the absence of the twin tips enhanced the stable workability of the alloy.

Figure 7b shows the interaction of the dislocation and the γ’ precipitate at the cooling process. The magnification of the red rectangular region of Figure 7b enabled one to observe several parallel dislocations pileup around the γ/γ’ interface. Accordingly, it is believed that γ’ precipitate could generate more dislocations due to its interaction with dislocation during slow cooling.

#### 4.2.3. Grain Coarsening Inhibited by γ’ Precipitates in Grain Boundaries

**Figure 8:**
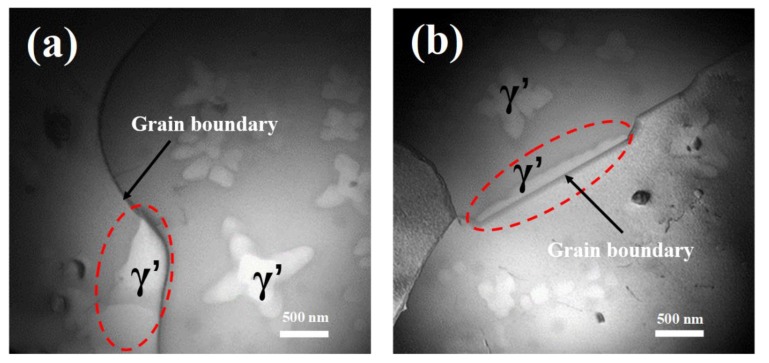
TEM images of FGH96 alloy (after cooling to 1000 °C with a cooling rate of 6 °C/min) exhibit γ’ precipitates in grain boundaries (marked by red dot circles) (**a**) the γ’ precipitate at the curving grain boundary; (**b**) the γ’ precipitate at the straight grain boundary.

Comparing with nucleation in the grains, γ’ precipitates preferred to nucleate and grow in the grain boundaries, since grain boundary was a fast channel for the diffusion of γ’ forming elements [43]. More importantly, precipitates in grain boundaries played an important role in inhibiting grain coarsening [26]. Figure 8 shows that the morphology of γ’ precipitates in grain boundaries was highly influenced by the orientation of grain boundaries and was significantly different to the dendritic γ’ precipitates in the interior of grains. Besides, it is clear from Figure 8 that the boundaries were not only the grain boundaries but also the phase boundaries, since γ’ precipitates could only exist in one of the two adjacent grains because of their coherent nature with the matrix. As a result, the phase boundary energy could also exist in the grain boundaries, which acted to inhibit the motion of the boundaries and improve the grain refinement during cooling process. Additionally, the long strip shape of γ’ precipitates, which increased the length of the phase boundaries, was an advantage for inhibiting grain coarsening and improving grain refinement.

## 5. Conclusion

The microstructural evolution of a polycrystalline nickel-base superalloy during pre-hot-deformation and subsequent slowly cooling was investigated. Grain refinement and homogeneous microstructure were achieved, and the underlying mechanism for these transitions was discussed. The conclusions can be drawn as follows:

(1) At 1150 °C, the steady flow stress occurred under a strain of 0.2 due to an excellent balance of work hardening and DRV/DRX softening, whereas a 14% decrease of stress occurred at the strain of 0.5.

(2) After pre-hot-deformation, it was found that twins mainly existed in large grains due to stress concentration. Twins hindered the motion of the dislocations during pre-hot-deformation, resulting in high dislocation density in large grains in which twinning occurred easily.

(3) When cooling to 1000 °C, the twin boundaries propagated through the entire grain, showing the absence of the twin tips inside the grains after cooling, whereas, in the initial stage of cooling, i.e., pre-hot-deformation to a strain of 0.5, plenty of twin tips were exhibited in the grains. In addition, the interaction of dislocations and γ’ precipitates in the course of cooling could generate pileup dislocations.

(4) The grain refinement during cooling process could be realized via (i) the occurrence of static recrystallization at grain boundaries by transforming large grains into plenty of small SRXed grains, (ii) the interaction of twin tips and γ’ precipitates by rotating γ’ precipitates to generate high angle grain boundaries, and (iii) coarse and irregular γ’ precipitates existing in the grain boundaries by hindering the coarsening of grains.

(5) Different from the intragranular γ’ precipitates with dendritic shape, the morphology of γ’ precipitates in grain boundaries (GB) was determined by orientation of GB, and the size of γ’ precipitates was much larger than intragranular γ’ precipitates due to the high diffusion rate of γ’ forming elements in GB. Because of coherent nature with the matrix, γ’ precipitates in GB could only exist in one of the two adjacent grains.

Grain refinement using the pre-hot deformation and subsequent cooling in this study could lead to a large amount of grain boundaries, including twin boundaries and high angle grain boundaries, which effectively hinder the motion of dislocations, thereby increasing the strength and corrosion resistance of the superalloy.

## Figures and Tables

**Table 1:** Chemical composition of FGH96 (wt.%).

C	Cr	Co	Mo	W	Al	Ti	Nb	B	Zr	Ni
0.030	16.000	13.000	4.000	4.000	2.200	3.700	0.800	0.011	0.036	Bal.

**Table 2:** Schmid factor of the twelve fcc slip systems for grain A.

Slip System	$\left(11\bar{1}\right)$			$\left(1\bar{1}1\right)$			$\left(1\bar{1}\bar{1}\right)$			(111)		
	[101]	[011]	$\left[1\bar{1}0\right]$	[110]	[011]	$\left[10\bar{1}\right]$	[101]	[110]	$\left[01\bar{1}\right]$	$\left[10\bar{1}\right]$	$\left[0\bar{1}1\right]$	$\left[1\bar{1}0\right]$
Schmid factor	0.24	0.33	0.08	0.08	0.33	0.24	0.49	0.16	0.33	0.49	0.33	0.16
